# Separation of Hepatic Iron and Fat by Dual-Source Dual-Energy Computed Tomography Based on Material Decomposition: An Animal Study

**DOI:** 10.1371/journal.pone.0110964

**Published:** 2014-10-30

**Authors:** Jing Ma, Zhi-Qiang Song, Fu-Hua Yan

**Affiliations:** 1 Department of Radiology, Shanghai Ruijin Hospital, Shanghai Jiao Tong University, Shanghai, China; 2 Department of Radiology, Shanghai Zhongshan Hospital, Shanghai Medical College, Fudan University, Shanghai, China; 3 Department of Radiology, the 2^nd^ Affiliated Hospital of Shi-Hezi University Medical School (Corps Hospital), Urumqi, Xinjiang Province, China; Institute of Automation, Chinese Academy of Sciences, China

## Abstract

**Objective:**

To explore the feasibility of dual-source dual-energy computed tomography (DSDECT) for hepatic iron and fat separation in vivo.

**Materials and Methods:**

All of the procedures in this study were approved by the Research Animal Resource Center of Shanghai Ruijin Hospital. Sixty rats that underwent DECT scanning were divided into the normal group, fatty liver group, liver iron group, and coexisting liver iron and fat group, according to Prussian blue and HE staining. The data for each group were reconstructed and post-processed by an iron-specific, three-material decomposition algorithm. The iron enhancement value and the virtual non-iron contrast value, which indicated overloaded liver iron and residual liver tissue, respectively, were measured. Spearman's correlation and one-way analysis of variance (ANOVA) were performed, respectively, to analyze statistically the correlations with the histopathological results and differences among groups.

**Results:**

The iron enhancement values were positively correlated with the iron pathology grading (r = 0.729, p<0.001). Virtual non-iron contrast (VNC) values were negatively correlated with the fat pathology grading (r = −0.642,p<0.0001). Different groups showed significantly different iron enhancement values and VNC values (F = 25.308,p<0.001; F = 10.911, p<0.001, respectively). Among the groups, significant differences in iron enhancement values were only observed between the iron-present and iron-absent groups, and differences in VNC values were only observed between the fat-present and fat-absent groups.

**Conclusion:**

Separation of hepatic iron and fat by dual energy material decomposition in vivo was feasible, even when they coexisted.

## Introduction

Coexisting hepatic iron deposition and steatosis can be associated with numerous liver diseases, such as fatty liver diseases, chronic hepatitis C and B, hepatocellular carcinoma, hemochromatosis and hemosiderosis [1], [2]. Qualitative and quantitative evaluation of hepatic iron and fat not only can provide more detailed diagnostic information, but such evaluations can also guide further medical treatment and management [3]–[6].

Currently, CT and magnetic resonance imaging (MRI) are widely used for the noninvasive detection of liver iron and fat in clinical practice, with each modality offering its own advantages and disadvantages [7]–[10]. MRI has limitations, including the overestimation of liver iron, underestimation of the fat percentage and the inability to quantify severe iron accumulation (e.g., more than 300 µmol/g), owing to the strong paramagnetic effects of iron, resulting in signal loss. Furthermore, the dependence on MRI protocol parameters and local field inhomogeneity preclude the widespread use of MRI [11]–[12]. Hepatic iron or fat can be assessed by traditional CT. Iron deposits increase the CT value of the liver parenchyma, while the opposite relationship is true for liver fat. If liver iron and fat coexist, then quantification of the fat or iron by conventional, single energy CT is no longer reliable due to the inverse effects of the iron and fat [13]. Previous studies have shown that the false negative rate of single energy CT on iron deposition was as high as 40% [14]. The accuracy of conventional CT in the diagnosis of fatty liver was approximately 67% in the absence of iron deposition and only 20% when there was iron deposition [15].

Compared to conventional single energy CT, dualenergy CT (DECT), particularly dual source DECT (DSDECT) equipped with two x-ray tubes and detectors, could simultaneously be used to acquire dual energy data with two different tube voltages, allowing for material decomposition with comparable atomic numbers due to the differences in the photon and Compton effects of CT values at different energies. Iodine, as a major contrast agent in clinical CT, has greater attenuation differences at low and high tube voltages, which could be easier to extract from other materials. Based on this principle, the original three-material decomposition algorithm of DSDECT for liver suggested that each voxel has only fat, liver tissue and iodine contrast agent. The attenuation difference for each material at the low and high tube voltages is very different as well. By the application of a complicated algorithm, iodine could be extracted as an iodine enhancement value (displayed as a colored image), and the fat and liver tissue are together measured as virtual non-enhanced CT values. Iron is similar to iodine, which also has greater attenuation differences at two different tube voltages. Iron could also be extracted with iron-specific calculation parameters. The iron enhancement value could specifically predict iron concentrations. The value greater than zero indicates presence of iron. The greater value means heavier iron deposition. In this manner, the virtual non-enhanced CT value, which is actually a virtual non-iron contrasted value (VNC value), could represent other liver tissues, mainly reflecting the degree of fat deposition. The smaller VNC value means heavier fat deposition.

Several in vitro experiments have quantified iron content by DECT, using an iron-specific, three-material decomposition algorithm [16]–[17]. In these in vitro studies, the VNC value was not explored. In our study, both the iron enhancement and liver parenchyma VNC values were investigated and correlated with pathology to assess the feasibility and reliability of DSDECT for hepatic iron and fat separation in vivo.

## Materials and Methods

All of the procedures in this study were approved by the Research Animal Resource Center of Shanghai Ruijin hospital. Sixty Wistar rats (male, 4 weeks old, weight 98–110 g) were fed by different diets and methods. All of the rats were anesthetized with an intraperitoneal injection of 0.3 ml/100 g of 10% chloral hydrate and were examined on a DSDECT system (SOMATOM Definition Flash, Siemens Medical Solutions, Forchheim, Germany). Unenhanced dual energy CT scanning was performed with tube voltages at 80 kVp and Sn140 kVp with a tin filter. The product of the tube current and the exposure time were set to 323 mA for the low voltage tube and 96 mA for high voltage tube; the detector collimation was 64*0.6 mm with 500 ms of gantry rotation time and 0.6 as a pitch value. The field of view (FoV) was 86 mm. An automated tube modulation system (CareDose4D; Siemens Medical Solutions) was used to minimize radiation exposure. The images were reconstructed with a 3 mm slice thickness and 2 mm slice interval, and the reconstruction kernel was D30. Then, the rats were sacrificed immediately to obtain pathology results. According to histological results (Perls' Prussian blue staining, hematoxylin and eosin), the rats were divided into the following four groups: normal group (total iron score [TIS] ≤15 and steatosis ≤5%); the fatty liver group (TIS≤15 and steatosis>5%); the coexisting liver iron and fat group (TIS>15 and steatosis>5%); and the iron group (TIS>15 and steatosis ≤5%).

*Annotation: The histological evaluation standard: A semi-quantitative evaluation of hepatic iron was performed according to the study by Deugnier et al [18]. Iron deposits were assessed according to the size and cellular and lobular locations in Rappaport's acinus, leading to three different scores: hepatocytic (HIS; range, 0–36); sinusoidal (SIS; range, 0∼12); and portal iron (PIS; range, 0∼12). The sum of these scores is defined the total iron score (TIS; range, 0∼60). Hepatic iron was graded as no iron overload (TIS 0∼15), mild (TIS 16∼30), moderate (TIS 31∼45) or severe (TIS 46∼60). Hepatic steatosis was graded as follows [19]: 0, no steatosis (less than 5% of hepatocytes affected); 1, mild (5∼33% of hepatocytes affected); 2, moderate (33∼66% of hepatocytes affected); and 3, severe (more than 66% of hepatocytes affected)*.

We retrospectively investigated the raw DECT scan data of each group. An iron specific three-material decomposition algorithm (fat, soft tissue and iron) for the liver was used as follows: the CT values of the tissue and fat for each rat were obtained in the General Viewing function of Dual Energy software at 80 kVp and Sn140 kVp, respectively. The ROI for fat was placed under the kidney (the area of ROI was 12.6 ±1.4 mm^2^) and that for normal soft tissue was placed at the erector spine muscle (the area of ROI was 13.2±2.9 mm^2^). These four CT values (fat and tissue at 80 kVp and Sn140 kVp) were entered into the Dual Energy liver VNC software for normalization. The slope (1.9) for iron-specific material decomposition was used in this study [17]. Then, a virtual iron concentration (VIC) image for each rat was generated. A colored VIC image provided the CT values of iron enhancement, which were similar to iodine enhancement in the contrast-enhanced CT scanning. An iron enhancement value greater than 0 indicated that iron is present, and larger iron enhancement values indicated heavier iron deposition. Virtual non-iron contrast (VNC) values could also be obtained with VIC imaging, representing the liver parenchyma (without iron). The measurements for each rat were obtained independently by two radiologists (with 2 and 3 years of experience, respectively, in abdominal imaging), who were blinded to the information about the rats and the scan protocol. Two consecutive CT slices at the middle liver (middle lobe and left medial lobe) were chosen. The iron enhancement values and VNC liver parenchyma values were measured on the VIC images, by placing ROIs on the middle hepatic lobe, avoiding the main vessels and bile ducts. The size of each ROI was chosen to include as much of the lobe as possible; the area of the ROI was 28.5±6.4 mm^2^.

### Statistical analysis

The variables are described as the means ± standard deviations. Inter-observer agreements were tested with the intraclass correlation coefficient (ICC). The correlations between the iron enhancement value and histopathological iron, VNC values and histopathological fat percentages were statistically analyzed by Spearman's correlation. One-way ANOVA was performed for the iron enhancement values and VNC values among groups. Significant differences were considered when p<0.05. All of the data were analyzed by SPSS software (SPSS for Windows, version 17.0; SPSS Inc., Chicago, IL, USA).

## Results

### 1 Intra-observer agreement

The inter-observer agreement regarding iron enhancement values and VNC values was good. The ICC of the iron enhancement value was 0.935, and the ICC of the VNC value was 0.917.

### 2 Histological results

**Figure 1:**
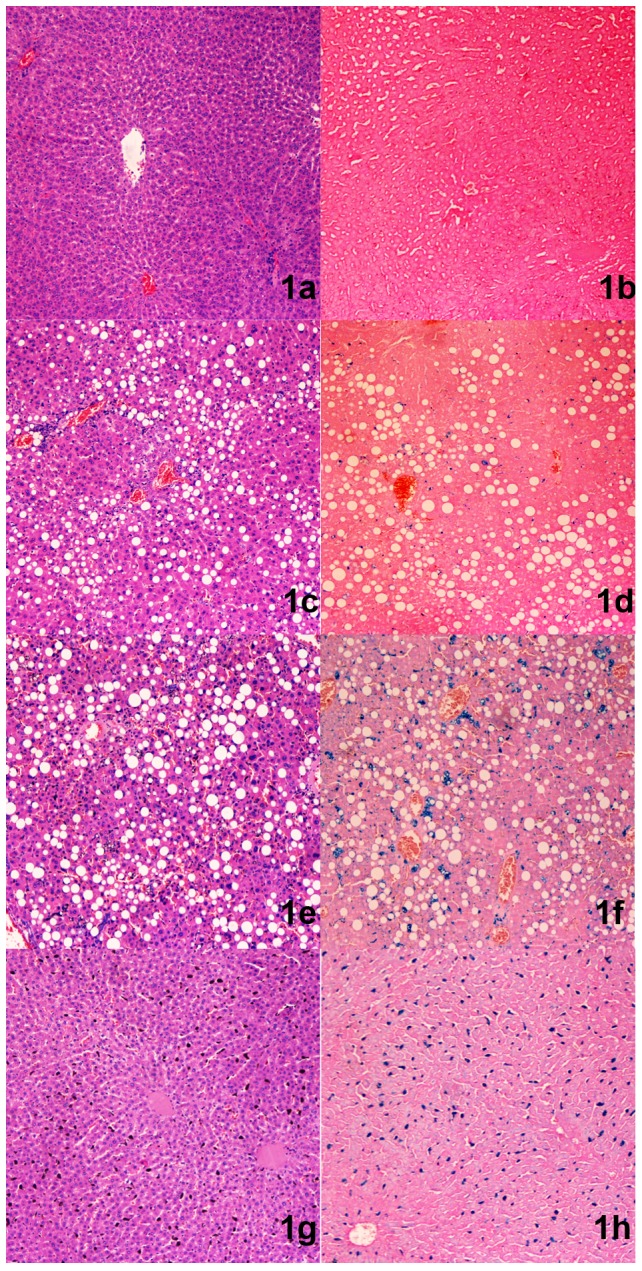
The rats were grouped by their liver histological results. Normal group: 1a) No lipid droplets in hepatocytes in the field of vision with HE staining. 1b) Prussian blue staining; there was no blue stained material in the field of vision, indicating the absence of iron deposition. Fatty liver group: 1c) Many hepatocytes were affected by lipid droplets on HE staining. 1d) Prussian blue staining resulted in no blue stained material, indicating the absence of iron deposition. Coexisting group: 1e) Many hepatocytes were affected by lipid droplets with HE staining. 1f) There were blue stained dots with Prussian blue staining, indicating significant iron deposition in this rat. Liver iron group: 1g) No hepatocytes were affected by lipid droplets on HE staining. 1h) There are blue stained dots in Prussian blue staining, indicating significant iron deposition in this rat.

The total liver iron grade scores of the 60 rats ranged from 5 to 57. Total steatosis measurements ranged from 1% to 90%. There were 5 rats in the normal group, 9 rats in the fatty liver group, 37 rats in the coexisting group and 9 rats in the liver iron group (Table 1). Rats in the normal group showed rare hepatic iron and steatosis (Figure 1a and 1b). In the fatty liver group, many hepatocytes with lipid droplets could be found with HE staining (Figure 1c), while the cells were negative for Perls' Prussian blue staining (Figure 1d). Both lipid droplets and blue dots were observed in the coexisting group (Figure 1e and 1f). In the liver iron group, the HE staining was negative (Figure 1g), but there were many blue dots with Perls' Prussian blue staining (Figure 1h).

**Table 1:** Histological results of 60 rats and groupings.

	Normal group	Fatty liver group	Coexisting fat and iron group	Liver iron group
	(TIS≤15and steatosis <5%)	(TIS ≤15 and steatosis ≥5%)	(TIS>15 and steatosis>5%)	(TIS>15 and steatosis <5%)
Number of rats in group	5	9	37	9
TIS (total iron score)	10±2	9±4	34±14	36±13
Steatosis (%)	3±2	47±33	50±27	4±1
Rat iron overload degree				
mild			15	4
moderate			10	2
severe			12	3
Rats steatosis degree				
1		4	12	
2		1	12	
3		4	13	

### 3 The relationships between the iron enhancement value and TIS and between the VNC value and degree of steatosis

**Figure 2:**
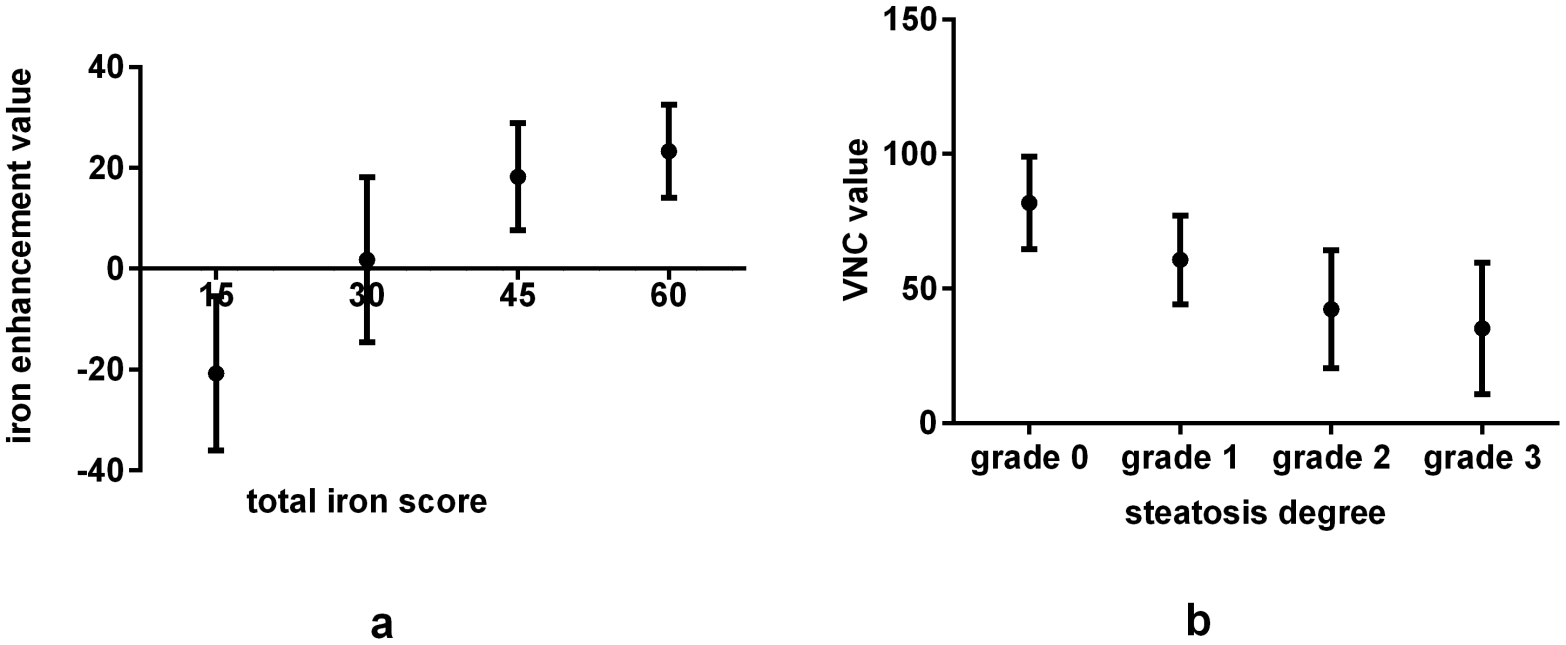
The iron enhancement value was increased with a larger TIS in the rats (r = 0.729, p<0.001)(a). The VNC values were decreased with a higher percentage of hepatocyte steatosis (r = −0.642,p<0.001) (b).

**Figure 3:**
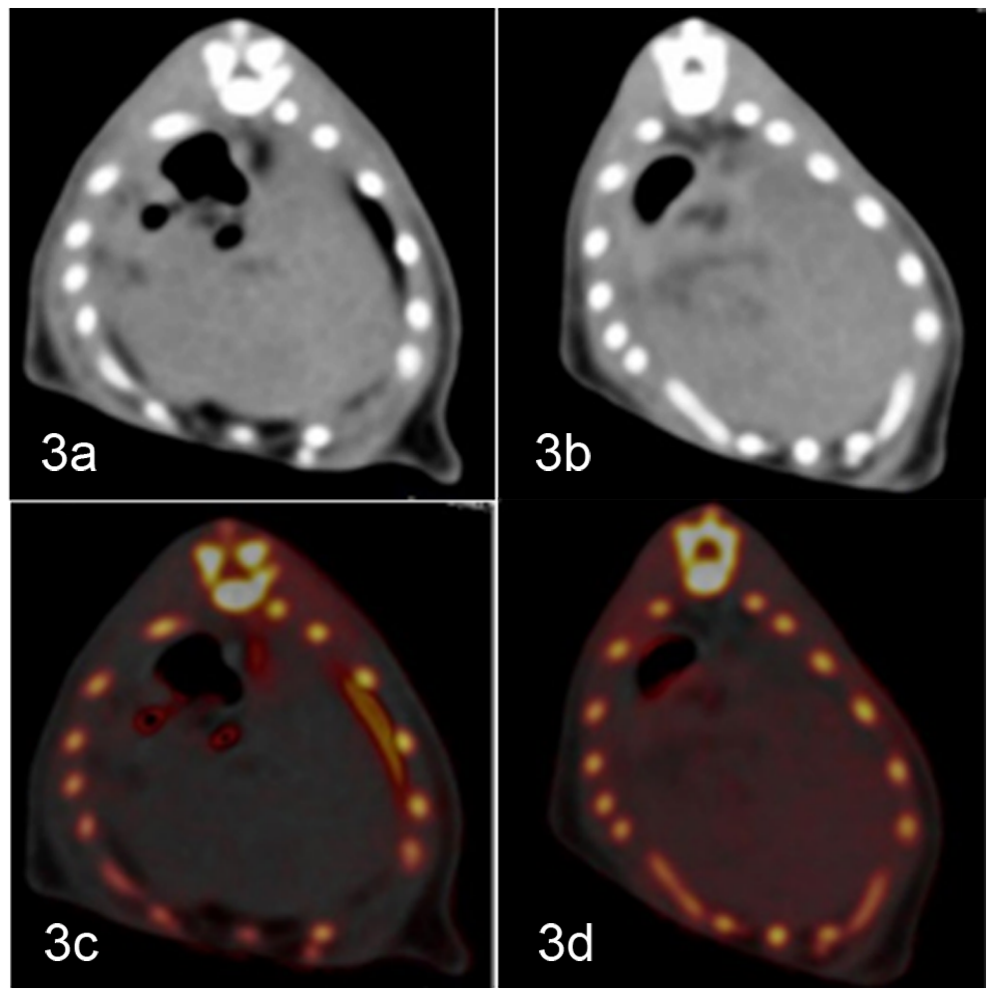
Examples showing mild and severe iron deposition; steatosis also coexisted in the liver. a–b) In the general view image, the degree of iron or steatosis in the liver could barely be determined from the gray-scale or CT value. c–d) On the VIC image, the existence and degree of iron are indicated in red colored dots and the iron enhancement value; the existence and degree of steatosis can be measured by the VNC value. For heavier iron deposition, more intense red colored dots and higher iron enhancement values were observed. More severe steatosis resulted in a smaller VNC value.

Iron enhancement values increased with increasing TIS (r = 0.729, p<0.001) (Figure 2a). The VNC values of the hepatic parenchyma decreased with increasing histological steatosis (r = −0.642, p<0.001) (Figure 2b). The iron distribution and concentration, represented by red dots, could be obviously visualized and measured on the VIC images (Figure 3).

### 4 Comparison of the iron enhancement values among groups

**Figure 4:**
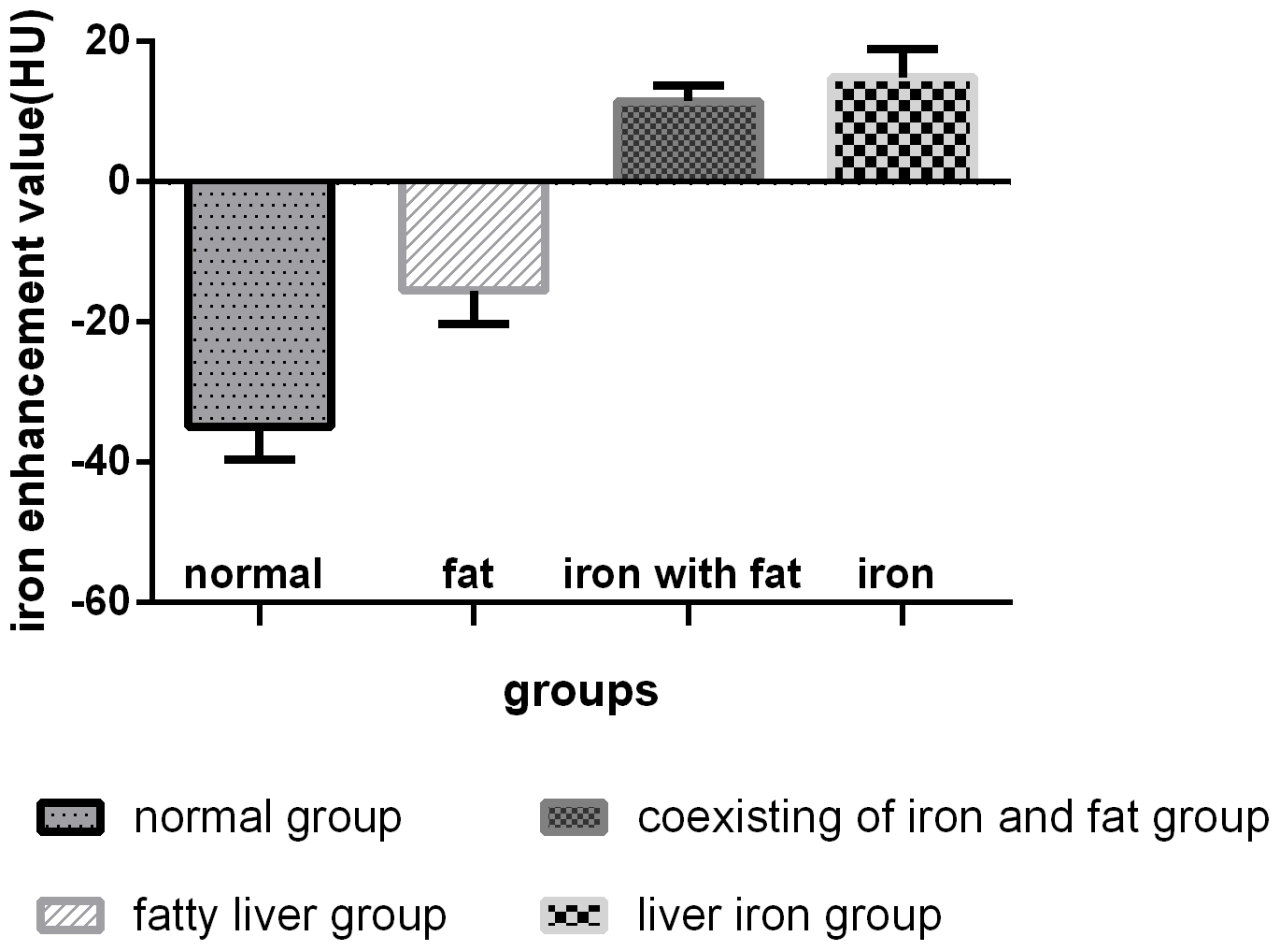
The iron enhancement values of the four groups were different among the groups (F = 25.308,p<0.001). The iron enhancement values for the iron-present groups (<0 HU) were significantly different from those of the iron-absent groups (>0 HU).

**Figure 5:**
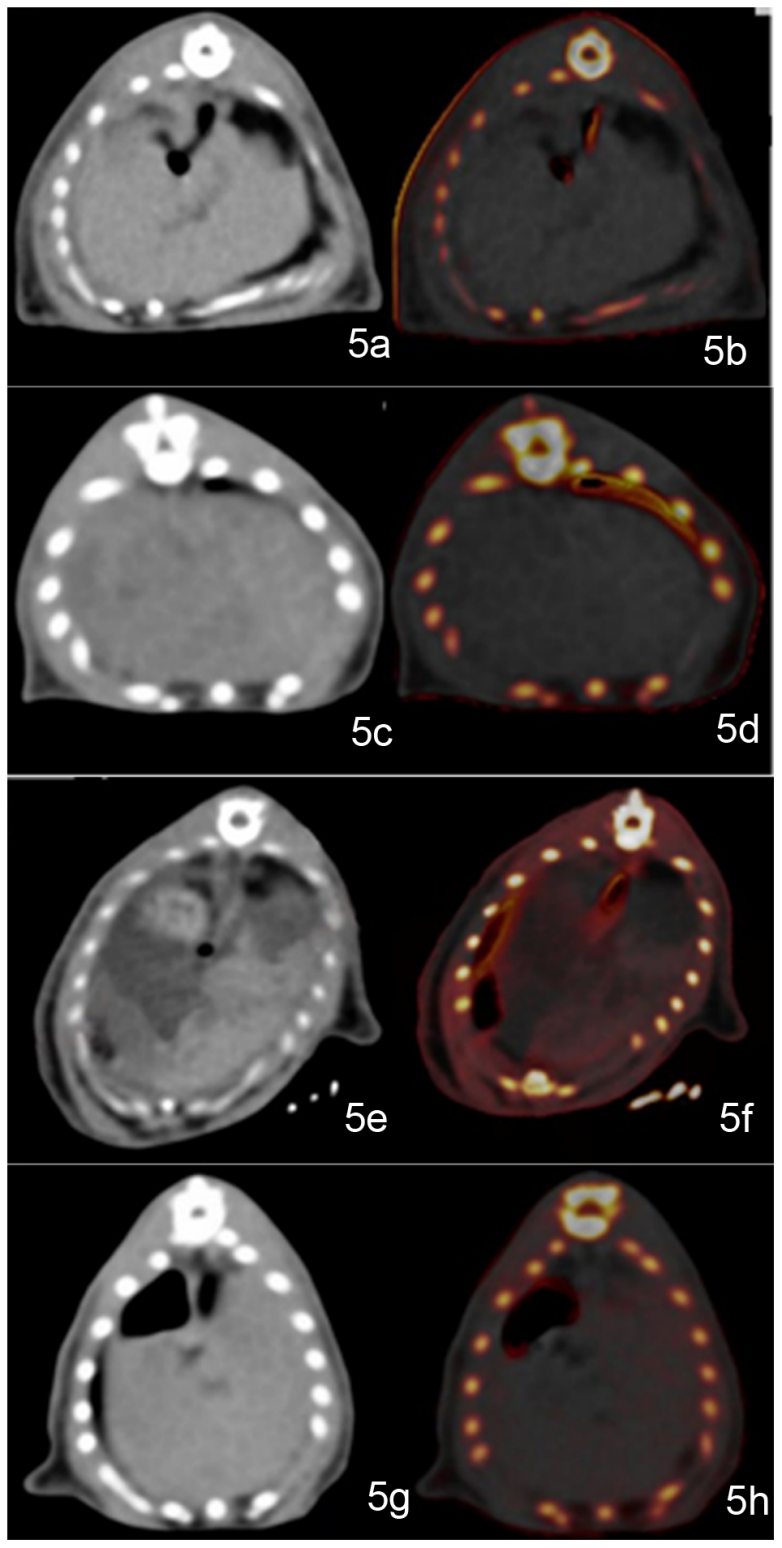
Example of a normal group rat liver. The rat liver periphery is outlined with a black line. The attenuation of the normal rat liver on a general view is homogeneous (CT value is 68 HU). On the VIC image, there are no red dots to show iron (iron enhancement value is −21.31 HU, VNC value is 71.85 HU) (a–b). Example of a rat in the fatty liver group. The rat liver on a general view had mildly lower density (CT value is 52.78 HU). On the VIC image, there are no red dots for iron (iron enhancement value is −13.20 HU, VNC value is 50.22 HU) (c–d). A typical image of a rat in the coexisting iron and fat group. Coincidentally, the dominant iron deposition region (outlined) is separated from the steatosis infiltration dominant region (*). The rat liver on a general view is inhomogeneous. Regions showing higher density might have been caused mainly by iron deposition (CT value is 83.72 HU). Regions showing lower density might be dominated by fat infiltration (CT value is 47.84 HU). On the VIC image, the red regions represent the presence of iron, corresponding to high-density regions on a general view image (iron enhancement value is 23.42 HU) (e–f). The color-absent region is also in accordance with the low-density region on the general view image (VNC value is 43.58 HU). Example of a rat in the liver iron group. The rat liver on general viewing showed mildly higher density (CT value is 80.35 HU). On the VIC image, there are scattered red dots that represent the presence of iron (iron enhancement value is 4.27 HU; VNC value is 72.35 HU) (g–h).

The iron enhancement values were significantly different among the groups (F = 25.308, p<0.001) (Figure 4). The following significant differences in iron content were observed: normal group *vs* coexisting group (p<0.001), normal group *vs* liver iron group (p<0.001), fatty liver group *vs* coexisting group (p<0.001), and fatty liver group *vs* liver iron group (p<0.001). The iron enhancement values of the iron-present groups were larger than 0 Housefield units (HU), while the absent groups were negative (<0 HU). Iron presence was visible on VIC imaging for both the liver iron and coexisting groups (Figure 5).

### 5 Comparison of VNC values among groups

**Figure 6:**
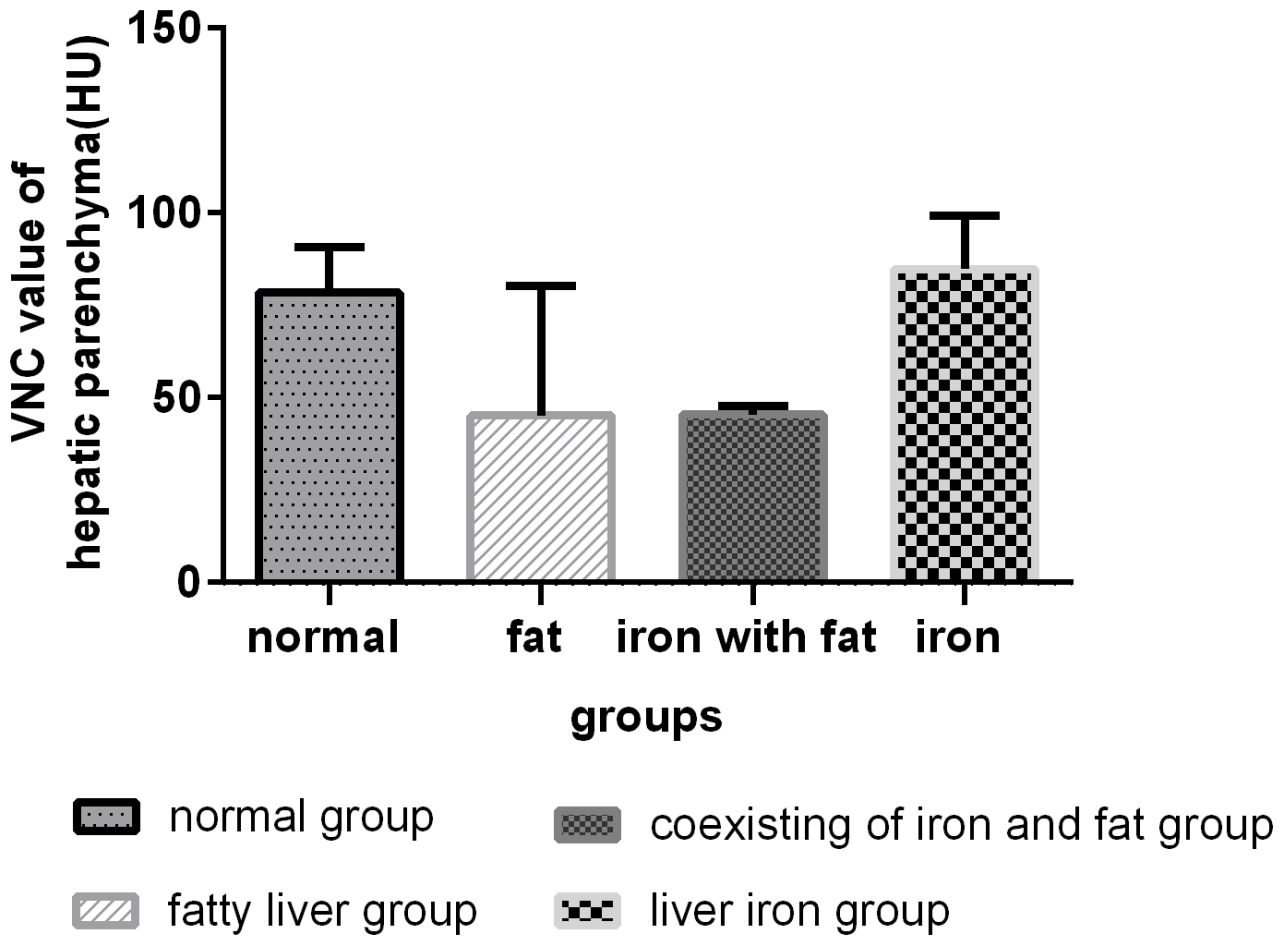
VNC values for the four groups were significantly different among the groups (F = 10.911,p<0.001). The VNC values for the steatosis-present groups were smaller than those for steatosis-absent groups.

The VNC values were also significantly different among the groups (F = 10.911, p<0.001) (Figure 6). The VNC values for the steatosis-present groups were smaller than for the steatosis-absent groups. The following significant differences were observed: normal group *vs* fatty liver group (p = 0.035), normal group *vs* coexisting group (p = 0.01), fatty liver group *vs* liver iron group (p<0.001), and coexisting group *vs* liver iron group (p<0.001).

## Discussion

Coexisting hepatic iron deposition and steatosis constitutes a common phenomenon in diffuse liver diseases [20]–[22]. Both single energy CT and MR have limited utility in qualitative and quantitative diagnosis when liver fat and iron coexist [8]. We designed a pilot study to separate rats' liver iron and fat using an iron-specific, three-material decomposition DECT algorithm. This study verified that DSDECT was feasible in vivo to separate liver iron from fat and that the imaging was correlated well with the pathological results for both iron and fat.

Our study was not the only one based on the DECT technology and algorithm; recent studies have used similar technology. Fischer MA et al quantified liver iron content with coexisting fat and also quantified liver fat in the presence of iron and iodine in their ex vivo study with dual-energy CT [16], [17]. In addition, Joe assessed the hepatic iron in liver phantoms and liver transplant recipients by calculating the CT attenuation (HU) difference between high and low tube voltages with DECT [23]. Although they were correlated well with the pathology in their study, however, we believe that the CT value differences in that study reflected mixed information, because the CT values at each tube voltage showed attenuation for both iron and other liver parenchyma (e.g., fat in the liver).

In our study, an iron-specific, three-material decomposition algorithm was used for iron quantification. The iron content was represented by the iron enhancement value and was presented using color VIC imaging. The iron enhancement values were positively correlated with histological iron. This value should not have been limited by the heavier iron deposition degree (TIS greater than 45), which was superior to MR. The differences in iron enhancement values among the four groups were significant, particularly between the iron-present (liver iron group and coexisting fat and iron group) and iron-absent groups (normal group and fatty liver group). The iron enhancement value could specifically reflect the iron content without fat interference. The results were in agreement with the in vitro experiments performed by Fischer MA. Our in vivo study brought us closer to the application of the iron enhancement value for iron quantification in the clinic. This method could provide more accurate liver iron quantification in diffuse liver disease.

The VNC values were negatively correlated with steatosis and also significantly different among different groups. Significant differences were observed between the fat-present (fatty liver group and coexisting fat and iron group) and fat-absent groups (normal group and liver iron group). Because the iron was subtracted with the three-material decomposition algorithm, the VNC value is sensitive to fat in liver. The VNC value is mainly determined by the steatosis degree and iron does not interfere with it. The VNC value has not previously been explored in any other studies. We demonstrated an added value of DECT, especially for fatty liver disease, which is usually accompanied by other liver disease. It is should be noted that the VNC value was influenced by the presence of other materials.

There were several limitations of this study. First, although the feasibility and reliability of DSDECT for iron and fat quantification were verified, this study was based on an in vivo animal model, and the iron or fat deposition patterns might be different in humans. In the future, patients with coexisting iron and fat in the liver should be investigated. Second, the VNC value was used to represent the degree of fat deposition in this study, while the VNC value was influenced by other liver parenchyma attenuation. Further technologies should be applied to predict the fat deposition more accurately.

In summary, the separation of hepatic iron from fat by DSDECT material decomposition in vivo was feasible, even when iron and fat coexisted.
